# NMR-based metabolomics identifies patients at high risk of death within two years after acute myocardial infarction in the AMI-Florence II cohort

**DOI:** 10.1186/s12916-018-1240-2

**Published:** 2019-01-07

**Authors:** Alessia Vignoli, Leonardo Tenori, Betti Giusti, Panteleimon G. Takis, Serafina Valente, Nazario Carrabba, Daniela Balzi, Alessandro Barchielli, Niccolò Marchionni, Gian Franco Gensini, Rossella Marcucci, Claudio Luchinat, Anna Maria Gori

**Affiliations:** 1 Magnetic Resonance Center (CERM), University of Florence, Sesto Fiorentino, Italy; 2 Consorzio Interuniversitario Risonanze Magnetiche di Metallo Proteine - C.I.R.M.M.P, Sesto Fiorentino, Italy; 3 Department of Experimental and Clinical Medicine, University of Florence, Florence, Italy; 4 Careggi Hospital, Florence, Italy; 5 Giotto Biotech S.r.l, Sesto Fiorentino, Florence Italy; 6 Unit of Epidemiology, ASL 10, Florence, Italy; 7 Centro Studi Medicina Avanzata (CESMAV), Florence, Italy; 8 Department of Chemistry, University of Florence, Sesto Fiorentino, Italy

**Keywords:** Acute myocardial infarction, Nuclear magnetic resonance, Serum, Metabolomics, Biomarker, Prognosis, Precision medicine

## Abstract

**Background:**

Risk stratification and management of acute myocardial infarction patients continue to be challenging despite considerable efforts made in the last decades by many clinicians and researchers. The aim of this study was to investigate the metabolomic fingerprint of acute myocardial infarction using nuclear magnetic resonance spectroscopy on patient serum samples and to evaluate the possible role of metabolomics in the prognostic stratification of acute myocardial infarction patients.

**Methods:**

In total, 978 acute myocardial infarction patients were enrolled in this study; of these, 146 died and 832 survived during 2 years of follow-up after the acute myocardial infarction. Serum samples were analyzed via high-resolution ^1^H-nuclear magnetic resonance spectroscopy and the spectra were used to characterize the metabolic fingerprint of patients. Multivariate statistics were used to create a prognostic model for the prediction of death within 2 years after the cardiovascular event.

**Results:**

In the training set, metabolomics showed significant differential clustering of the two outcomes cohorts. A prognostic risk model predicted death with 76.9% sensitivity, 79.5% specificity, and 78.2% accuracy, and an area under the receiver operating characteristics curve of 0.859. These results were reproduced in the validation set, obtaining 72.6% sensitivity, 72.6% specificity, and 72.6% accuracy. Cox models were used to compare the known prognostic factors (for example, Global Registry of Acute Coronary Events score, age, sex, Killip class) with the metabolomic random forest risk score. In the univariate analysis, many prognostic factors were statistically associated with the outcomes; among them, the random forest score calculated from the nuclear magnetic resonance data showed a statistically relevant hazard ratio of 6.45 (*p* = 2.16×10^−16^). Moreover, in the multivariate regression only age, dyslipidemia, previous cerebrovascular disease, Killip class, and random forest score remained statistically significant, demonstrating their independence from the other variables.

**Conclusions:**

For the first time, metabolomic profiling technologies were used to discriminate between patients with different outcomes after an acute myocardial infarction. These technologies seem to be a valid and accurate addition to standard stratification based on clinical and biohumoral parameters.

**Electronic supplementary material:**

The online version of this article (10.1186/s12916-018-1240-2) contains supplementary material, which is available to authorized users.

## Background

Among cardiovascular diseases (CVDs), acute coronary syndrome (ACS) represents the most common cause of emergency hospital admission and it is associated with the highest mortality and morbidity [1, 2]. The prognosis is directly associated with timely initiation of revascularization, and misdiagnosis or late diagnosis may have unfavorable clinical implications. Established risk stratification tools such as the Global Registry of Acute Coronary Events (GRACE) and the Thrombolysis In Myocardial Infarction risk scores are derived from demographic, clinical, laboratory, and electrocardiogram-related variables [3, 4]. These do not incorporate the use of newer biomarkers, which could represent different pathophysiologic processes and provide complementary prognostic information, thereby improving risk stratification beyond traditionally used variables.

Several studies have evaluated the potential clinical usefulness of new biomarkers able to identify patients who had a poor outcome. In particular, high levels of inflammatory markers such as C-reactive protein and interleukin-8 had long-term prognostic utility in patients with ACS that undergone coronary revascularization [5, 6]. However, no conclusive and consistent data about the prognostic utility of measuring inflammatory markers in the early phase of ACS are available in the literature.

A number of studies have evidenced that a global approach, such as genomics, proteomics, or metabolomics, may represent a valid strategy for improving current knowledge about pathophysiological mechanisms and for identifying ACS patients at high risk of secondary atherothrombotic events or premature death.

Metabolomics is the accepted name for the -omic science that deals with the characterization of the metabolome, in turn defined as the whole set of metabolites in a certain biological system, such as a cell, tissue, organ, or entire organism [7]. The two leading analytical techniques used to perform metabolomics are mass spectrometry (MS) and nuclear magnetic resonance (NMR) spectroscopy. Both techniques yield information about many different molecules in a single measurement, and can be used to determine structures and concentrations of metabolites [8]. Nevertheless, each technique has its own strengths and limitations. MS overshadows NMR in terms of numbers of compounds resolved (of the order of 10^3^ [9]), with a sensitivity down to the picomolar and requiring a very small volume of the biospecimen; however, reproducibility is still a limitation of MS, which must be overcome by an extensive and time-consuming use of standards and quality control samples. NMR analysis is high-throughput [7], and NMR data are highly reproducible [10] and intrinsically quantitative over a wide dynamic range, as demonstrated by numerous ring trials performed by many different NMR laboratories [10]. NMR gives immediately qualitative and quantitative information on around 10^2^ different small molecules present in a biological sample [11], and has already provided a global picture of a wide range of metabolic processes underlying complex and multifactorial diseases such as ACS.

Recently, the metabolomic approach has been applied to identify a risk profile in heart failure patients [12–14], atrial fibrillation patients [15], and diabetic patients [16]. In the setting of ACS, studies have characterized the metabolic biosignature of myocardial ischemia [9, 17–20], identified altered signatures in lipid metabolism in patients with angina or myocardial infarction with respect to control subjects [21], and identified microbial metabolites in urine associated with coronary heart disease [22].

Risk stratification should identify individuals at high risk who require more intensive therapy, or, conversely, help avoid drug overuse and associated side effects in patients with a favorable prognosis. In this framework, the aim of the present study was to evaluate the impact of the metabolomic fingerprint on the occurrence of cardiovascular death in acute myocardial infarction (AMI) patients after percutaneous coronary intervention.

## Methods

This study was part of a collaborative project between the Department of Medical and Surgical Critical Care of the University of Florence and the Magnetic Resonance Centre (CERM) of the University of Florence.

### Study population

The study population comprised 978 out of 1496 patients admitted to the coronary units of the six hospitals (five community hospitals and one university hospital, the Careggi Hospital) of the Florence health district between April 2008 and April 2009, and enrolled in the frame of the Florence Acute Myocardial Infarction-2 (AMI-Florence 2) registry [23]. In the present study, we evaluated 978 patients (345 women and 633 men, median age 74 years); among them, 146 patients died within 2 years of the AMI event and 832 patients survived for at least 2 years. The 2-year vital status was assessed by consulting the registry office of the city of residence. Mortality analysis was therefore censored at 24 months after AMI or at date of death, whichever occurred earlier. For this study, 35% of the AMI-Florence 2 population was excluded because of the lack of a good quality blood sample for the metabolomic analyses and/or because follow-up information was not available (Additional file 1: Figure S1). However, according to a standard power analysis [24] using a *t* test as the test statistic, and fixing an alpha level of 0.05 for a significant comparison, it was found that having 146 patients who died and 832 patients who survived was enough to detect small to medium effects (Cohen’s d ~0.25) with a statistical power of 80%; furthermore, the training and validation sets separately showed adequate statistical power (Cohen’s d = 0.54 and 0.29, respectively). Blood samples were collected 24–48 h after percutaneous coronary intervention (PCI) and overnight fasting. All information about inclusion criteria and treatment of the patients are detailed in Additional file 1: Supplementary material.

All subjects gave written informed consent. The study (number 11/2008) complies with the Declaration of Helsinki and was approved by the ethics committees of the local health unit, the University of Florence, and Careggi Hospital (19 March 2008).

### NMR analyses

Samples were prepared following the standard protocols detailed by Bernini et al. [25]. According to standard practice [26], all spectra were acquired at 310 K using a Bruker 600 MHz spectrometer (Bruker BioSpin), and for each serum sample three one-dimensional ^1^H-NMR spectra, namely nuclear Overhauser effect spectroscopy (NOESY), Carr–Purcell–Meiboom–Gill (CPMG), and Diffusion-edited spectra, were acquired, allowing the selective detection of different molecular weight metabolites. A detailed description of the sample preparation and experiments is presented in the Additional file 1: Supplementary material and Figure S1.

Each one-dimensional spectrum in the range 0.2–10.00 ppm was segmented into 0.02 ppm chemical shift bins and the corresponding spectral areas were integrated using AMIX software (version 3.8.4, Bruker BioSpin). The region between 4.5 and 5.0 ppm containing the residual water signal was removed and the dimension of the system was reduced to 466 bins. The total spectral area was calculated on the remaining bins and total area normalization was carried out on the data prior to pattern recognition.

### Statistical analysis

Data analyses were performed using the open source software R. For the demographic and baseline characteristics, the *t* test was used for comparison between groups and the chi-square test for comparison between categorical variables.

For the multivariate data analyses of the NMR data, the group of 978 patients was randomly split into two independent cohorts [27]: a training set constituting 80 patients who survived and 40 who died, and a validation set constituting all remaining patients (106 patients who died and 752 who survived). A prospective power analysis [24] was employed to determine the minimum number of patients (surviving and dead) that would need to be retained in the training set to have a sufficiently powered model, thereby maintaining a large fraction of samples for the validation to guarantee a reliable estimation of the performance of the model. After determining that 80 patients who survived and 40 who died should be retained in the training set, the allocation between training and validation was performed randomly.

The initial analysis was restricted to the training set and the first step was to establish if serum metabolomic profiles could distinguish between patients who survived and died within 2 years after the cardiovascular event. For this purpose, a random forest (RF) classifier [28] was built (considering for each sample the full spectrum; thus, no choice of particular metabolite was performed). The percentage of trees that assign one sample to a specific class can be inferred as a probability of class belonging [29–31]. For each patient, a score was created that expressed the extent to which the serum metabolomic profile appeared to be similar to the profile of one of the patients who died, designated as the ‘RF risk score’. For each patient, three RF scores were derived using the three types of spectra acquired. For all calculations, the R package ‘Random Forest’ [32] was used to grow a forest of 2,000 trees, using the default settings (see Additional file 1: Supplementary material for further details on the RF approach).

The next step was to test the hypothesis that a metabolomic signature similar to that of one of the patients who died would be predictive of death within 2 years after the cardiovascular event. Using receiver operating characteristics (ROC) analysis (“colAUC” function of the R package “caTools”) and Harrell’s c index (“cindex” function on the R package “dynpred”), the performances of the RF risk scores were compared with the actual outcome. To delineate high risk of death, a cut-off for the RF risk score was calculated in the training set that optimized accuracy, sensitivity, and specificity, and the performance of the model was subsequently tested in the validation set.

The performances of the NOESY RF score were evaluated by calculating a Cox proportional hazards regression model [33] using the function “coxph” (R package “Survival”) and the model significance was assessed through a likelihood-ratio test and by calculating the model concordance. The independent prognostic capacity of the RF risk score model in comparison with standard prognostic features was also evaluated using Cox models. The performance of NOESY RF scores was also compared with the performance of the GRACE score and with a linear combination of the two scores through ROC analysis, Harrell’s c index, and univariate Cox models.

The spectral regions related to 23 metabolites, present in concentrations above the detection limit (>1 μM) in all samples (up to 30–40 different metabolites could be quantified in each sample [34]), were assigned in the CPMG NMR spectra by using matching routines of AMIX 3.8.4 (Bruker BioSpin) in combination with the BBIOREFCODE database (Bruker BioSpin) and the freely available Human Metabolome DataBase [35], and quantified. Metabolite quantification was determined by software developed in-house based on standard line-shape analysis methods. Using this approach, each NMR region of interest was decomposed and deconvoluted into its component parts that corresponded to its number of protons, and then integrated to obtain the metabolite concentrations in arbitrary units (Additional file 1: Figure S2). Wilcoxon signed-rank test [36] was chosen to infer differences between the metabolites concentrations of the outcome groups on the biological assumption that metabolite concentrations are not normally distributed, and false discovery rate correction was applied using the Benjamini–Hochberg method [37]. An adjusted *P*-value < 0.05 was deemed significant. Effect size using Cliff’s delta [38] was calculated by means of the R package “effsize”.

The statistical approach described above was also used to build sex-specific statistical models based on NOESY spectra.

NMR and clinical data are freely available in the Open-Access Database Repository MetaboLights from October 2017 with the accession number MTBLS395 (http://www.ebi.ac.uk/metabolights).

## Results

Demographic and clinical characteristics of the enrolled patients are shown in Table 1. The characteristics of patients according to gender are also reported in Additional file 1: Table S1.

**Table 1:** Demographic and clinical characteristics

	Survived (*n*=832)	Died (*n*=146)	*p*-value
Demographic characteristics,			
Age (years), median (IQR)	72 (62–80)	82 (78–83)	< 2.20×10^−16^
Female sex, *n* (%)	278 (33.4)	67 (45.9)	4.86×10^−03^
Cardiovascular risk factors, n (%)			
Hypertension	537 (64.5)	104 (71.2)	1.21×10^−01^
Dyslipidemia	294 (35.3)	32 (21.9)	3.55×10^−03^
Current smokers	226 (27.2)	20 (13.7)	1.45×10^−04^
Ex-smokers	21 (2.5)	8 (5.5)	4.01×10^−01^
CAD	220 (26.4)	17 (11.6)	5.03×10^−04^
Diabetes	197 (23.7)	61 (41.8)	6.32×10^−06^
Medical history, n (%)			
Myocardial infarction	164 (19.7)	48 (32.9)	5.23×10^−04^
Angina, onset > 1 month	119 (14.3)	24 (16.4)	8.96×10^−01^
Angina, onset ≤ 1 month	149 (17.9)	15 (10.3)	3.78×10^−02^
CABG	41 (4.9)	10 (6.8)	4.39×10^−01^
PCI	136 (16.3)	32 (21.9)	1.20×10^−01^
Chronic heart failure	33 (4.0)	26 (17.8)	2.62×10^−10^
Atrial fibrillation	42 (5.0)	21 (14.4)	4.60×10^−05^
Cerebrovascular disease	50 (6.0)	28 (19.2)	1.28×10^−07^
Presentation features			
ACS classification, STEMI, n (%)	343 (41.2)	35 (24.0)	1.15×10^−04^
Killip II–IV, *n* (%)	114 (13.7)	61 (41.8)	6.33×10^−16^
Creatinine > 1.2 mg/dL, *n* (%)	129 (15.5)	54 (37.0)	4.08×10^−09^
Heart rate (bpm), median (IQR)	80 (67–91)	90 (80–105)	3.66×10^−06^
Positive peak troponine maximum, *n* (%)	804 (96.6)	143 (97.9)	6.98×10^−01^
Positive peak CK-MB maximum, *n* (%)	422 (50.7)	50 (32.2)	1.88×10^−03^
GRACE score > 118, *n* (%)	687 (82.6)	123 (84.2)	1.62×10^−01^

*CAD* coronary artery diseases, *CABG* coronary artery bypass grafting, *PCI* percutaneous coronary intervention, *ACS* acute coronary syndrome, *STEMI* ST-segment elevation myocardial infarction, *CK-MB* creatine kinase-MB, *GRACE* Global Registry of Acute Coronary Events

### NMR spectra

For each sample three NMR metabolomic profiles were obtained using NOESY, CPMG, and Diffusion-edited pulse sequences (Additional file 1: Figure S3). According to the peculiar characteristics of each NMR experiment, high and/or low molecular weight metabolites can be detected. An exploratory principal component analysis of the dataset is reported in Additional file 1: Figure S4.

### Discrimination of the outcomes and the RF scores in the training set

**Fig. 1:**
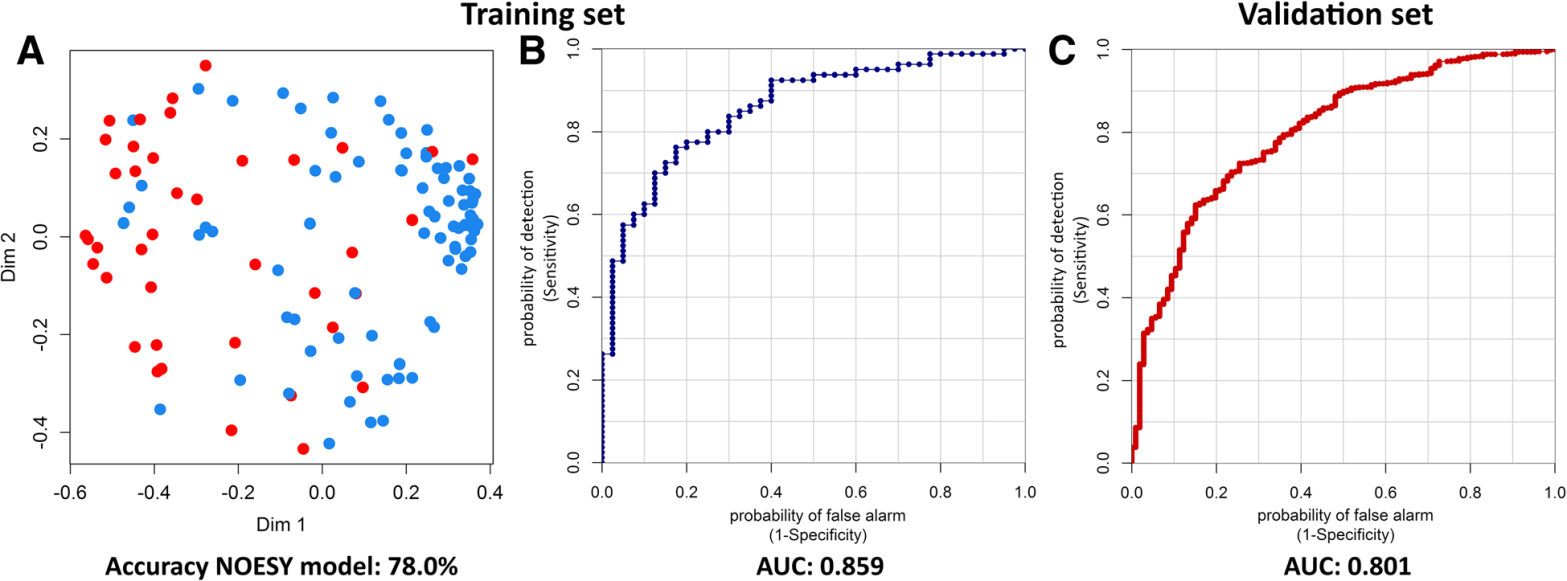
Clusterization of serum metabolomic profiles and comparisons between metabolomic classification and outcomes in the training set and the validation set. **a** Discrimination between patients who survived (blue dots, n = 80) and died (red dots, n = 40) using the Random Forest classifier on nuclear Overhauser effect spectroscopy (NOESY) spectra in the training set. **b, c** The receiver operator characteristic curves and the area under the curve (AUC) scores are presented for the training set (**b**) and validation set (**c**)

The metabolomic profiles of 80 patients who survived and 40 who died (training set) were classified using the RF classifier. The profiles showed significant differential clustering, with good separation of the two groups using each type of NMR spectra: NOESY (Fig. 1a), CPMG, and Diffusion. Using ROC analyses, the areas under the curve (AUC) obtained were 0.859 for NOESY spectra (Fig. 1b), 0.857 for CPMG spectra, and 0.775 for Diffusion editing spectra. NOESY and CPMG models showed approximately the same performances, and the discrimination between the two outcome groups can be ascribed to both low (mostly) and high molecular weight metabolites. Thus, it was decided to use the NOESY spectra in the further analyses because they contained both high and low molecular weight metabolite information.

A threshold of ≥ 0.454 for the NOESY RF score was set to optimize accuracy, sensitivity, and specificity; this optimized threshold yielded 76.9% (95% confidence interval (CI) 76.5–77.3%) sensitivity, 79.5% (95% CI 78.4–80.6%) specificity, and 78.2% (95% CI 77.6–78.7%) accuracy.

The goodness of fit of the NOESY RF model was calculated using a Cox proportional hazards regression model: NOESY RF showed a hazard ratio of 7.4 (95% CI 3.51–15.6) with a *p-*value of 3.45×10^−09^ calculated using the likelihood-ratio test, and a concordance coefficient of 0.73.

Moreover, the NOESY RF model was robust with respect to different strategies of model validation; indeed, a mean AUC of 0.805 (95% CI 0.795–0.814) was obtained with 100 cycles of classical Monte Carlo cross-validation with data split 80 to 20% (training set, validation set). Furthermore, our approach was robust with respect to the origin of samples: only slight differences in the AUC were obtained when using samples from the Careggi University Hospital as the training set to build the RF model and samples from all the other hospitals as the validation set (and vice versa) (Additional file 1: Figure S5).

### Outcome prediction by NOESY RF score in a validation set of patients

The validation set (106 patients who died and 752 who survived) was evaluated using an unsupervised analysis. Spectra of the validation samples were classified as either ‘dead’ or ‘survivor’ using the optimized NOESY RF risk score model derived from the training set. Comparison between metabolomic classification and actual outcome demonstrated high correlation with an AUC of 0.801 (Fig. 1c). Using the threshold maximized in the training set, we obtained 72.6% sensitivity, 72.6% specificity, and 72.6% overall predicting accuracy.

The AUC calculated on the RF score was assessed for significance against the null hypothesis of no prediction accuracy in the data by means of 10,000 randomized class-permutations tests: the estimated AUC obtained after randomization was 0.570 (95% CI 0.569–0.571), demonstrating the significance of our result (AUC 0.801, *p* = 2.21×10^−06^).

The performances of the NOESY RF model in the validation set was assessed using a Cox proportional hazards regression model: NOESY RF showed a hazard ratio of 6.16 (95% CI 4.02–9.44) with a *p-*value of 1.11×10^−16^ calculated using the likelihood-ratio test, and a concordance coefficient of 0.71.

### Comparison of the NOESY RF score with known prognostic factors and the GRACE score

The known prognostic factors age, sex, previous CABG, previous PCI, heart failure, atrial fibrillation, cerebrovascular disease, diabetes, creatinine concentration, Killip class, and ACS classification were compared with the NOESY RF risk score, calculated on the entire dataset, in univariate and multivariate Cox regression analyses. The results are displayed in Table 2.

**Table 2:** Association with the outcome: unadjusted and adjusted hazard ratios

	Hazard ratio (univariate)	*p*-value	Hazard ratio (multivariate)	*p*-value
Age				
68–79	4.63	2.24×10^−04^	2.09	1.45×10^−01^
> 79	12.86	8.90×10^−11^	3.87	8.33×10^−03^
Male sex	0.64	1.35×10^−02^	0.96	8.72×10^−01^
Hypertension	1.34	1.47×10^−01^	0.53	1.98×10^−02^
Dyslipidemia	0.55	7.16×10^−03^	0.38	7.91×10^−03^
Smoking habits				
Yes	0.43	1.67×10^−03^	1.25	5.04×10^−01^
Ex-smokers	1.13	7.95×10^−01^	4.14×10^-08^	9.95×10^−01^
CAD	0.41	2.00×10^−03^	0.99	9.86×10^−01^
Previous CABG	1.16	7.10×10^−01^	1.92	2.61×10^−01^
Previous PCI	1.28	2.69×10^−01^	1.66	9.64×10^−02^
Heart failure	3.67	1.07×10^−07^	1.81	8.98×10^−02^
Atrial fibrillation	2.37	7.03×10^−04^	1.30	4.52×10^−01^
Cerebrovascular disease	3.17	4.10×10^−07^	1.96	2.67×10^−02^
Diabetes	1.99	1.83×10^−04^	1.05	8.58×10^−01^
Creatinine (>1.2 mg/dL)	2.89	8.70×10^−09^	1.29	3.37×10^−01^
Killip class				
II	3.43	4.89×10^−09^	1.77	4.26×10^−02^
III	4.87	9.12×10^−10^	3.31	4.76×10^−04^
ACS classification				
STEMI	0.49	1.34×10^−03^	0.72	3.00×10^−01^
NOESY RF risk score				
≥ 0.454	6.45	2.16×10^−16^	3.71	2.36×10^−05^
GRACE score^#^				
≥ 170	6.05	3.76×10^−06^	–	–
NOESY RF + GRACE^#^				
≥ 7.7	9.33	2.16×10^−16^	–	–

Correlation with the outcome for prognostic features and RF risk score in the full dataset, using univariate and multivariate analysis. Age split into tertiles. In the multivariate, hazard ratios of all the variable were included together in the analysis

*CAD* coronary artery diseases, *CABG* coronary artery bypass grafting, *PCI* percutaneous coronary intervention, *ACS* acute coronary syndrome, *STEMI* ST-segment elevation myocardial infarction, *NOESY* nuclear Overhauser effect spectroscopy, *RF* random forest, *GRACE* Global Registry of Acute Coronary Events

^#^These variables were not included in the multivariate analysis due to the strong co-linearity between GRACE score and the other clinical variables [66]

In the univariate analysis, many prognostic factors were statistically associated with the outcomes, among them the NOESY RF score showed a Cox hazard ratio of 6.45. In the multivariate analysis, only age, dyslipidemia, hypertension, previous cerebrovascular disease, Killip class, and NOESY RF score remained statistically significant, demonstrating their independence with respect to the other variables. The smoker’s paradox, already described by other studies [39], was also observed in this cohort.

**Fig. 2:**
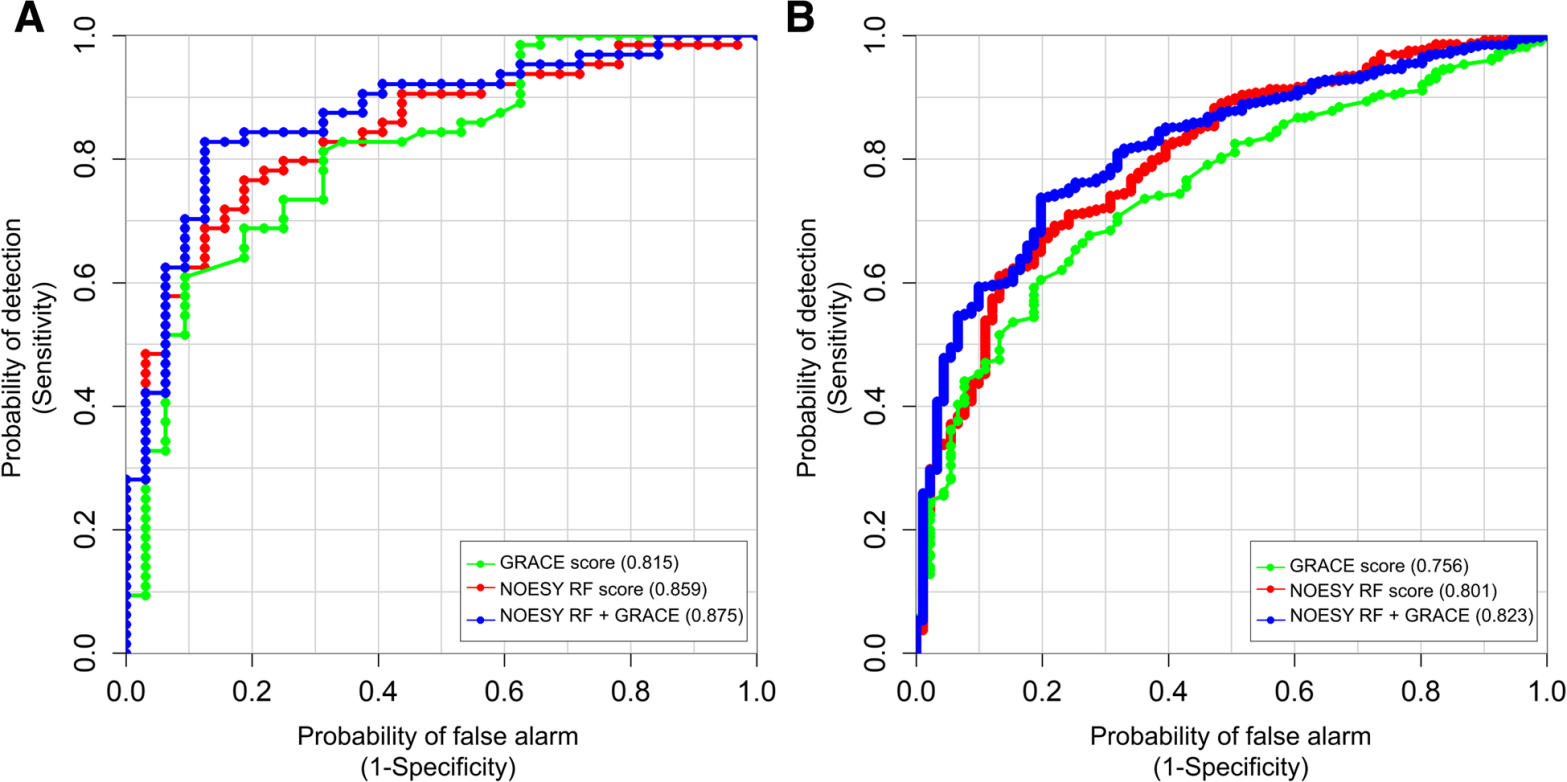
Receiver operator characteristic curve and area under the curve (AUC) of nuclear Overhauser effect spectroscopy (NOESY) random forest (RF) score, Global Registry of Acute Coronary Events (GRACE) score, and linear combined score of NOESY RF and GRACE score are reported for the **a** training and **b** validation sets

The performance of the NOESY RF score in predicting 2-year outcomes was also compared with the performance of the GRACE hospital discharge risk score [40] for those patients for whom all clinical parameters needed to calculate the GRACE score were available (84.5% of patients). The results (Harrell’s c index and AUC) demonstrated that the NOESY RF score performed slightly better in both the training and the validation sets (Table 3, Fig. 2), confirming that the metabolomic approach described here could be useful for risk stratification in the setting of post-AMI patients. Moreover, the score obtained by a linear combination of the two scores (NOESY RF + GRACE scores) was calculated (Table 3, Fig. 2); the combined model showed statistically improved results (analysis of variance test *p*-value < 0.01 after Bonferroni correction) with respect to the two separate scores in both the training and validation sets. A comparison of these two scores with the NOESY RF score is included in the Cox regression analyses in Table 2.

**Table 3:** Comparison between GRACE score, NOESY RF score and a linear combination of NOESY RF and GRACE scores (GRACE + NOESY RF scores)

	GRACE score	NOESY RF score	GRACE + NOESY RF scores
Training set			
AUC (95% CI)	0.815 (0.794-0.820)	0.859 (0.858-0.860)	0.875 (0.864-0.885)
Harrell’s c index	0.776 (0.761-0.781)	0.806 (0.805-0.809)	0.828 (0.821-0.835)
Validation set,			
AUC	0.756 (0.754-0.758)	0.801 (0.800-0.802)	0.823 (0.822-0.825)
Harrell’s c index	0.740 (0.744-0.747)	0.789 (0.789-0.790)	0.809 (0.808-0.810)

*GRACE* Global Registry of Acute Coronary Events, *NOESY* nuclear Overhauser effect spectroscopy, *RF* random forest, *AUC* area under the receiver operating characteristic, *CI* confidence interval

**Fig. 3:**
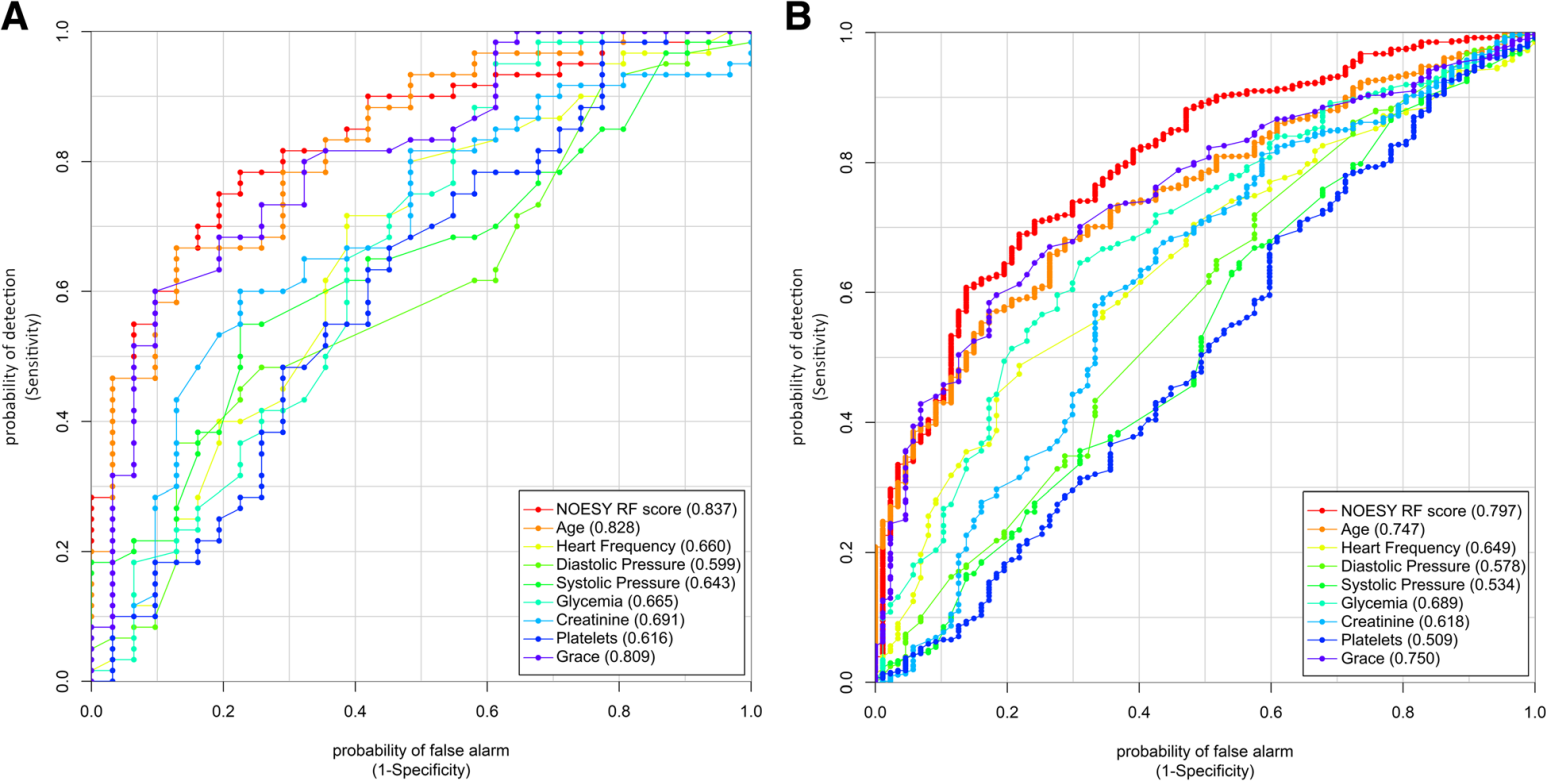
Nuclear Overhauser effect spectroscopy (NOESY) random forest (RF) score, Global Registry of Acute Coronary Events (GRACE) score, and clinical parameters receiver operator characteristic (ROC) curves for the **a** training and **b** validation sets. The ROC curves and the area under the curve scores are presented for NOESY RF score, GRACE score, age, heart frequency, diastolic pressure, systolic pressure, glycemia, creatinine, platelets, troponine maximum, and creatine kinase-MB maximum

Using ROC analyses, the performance of the NOESY RF and GRACE scores was tested against some well-known parameters that are commonly measured in the clinical practice (age, heart frequency, diastolic pressure, systolic pressure, creatinine concentration, glycemia, platelets). Training and validation sets were considered separately and only data from patients for whom all the clinical parameters were available were included (80.5% of the entire cohort). As shown in Fig. 3, our score showed the best performances in predicting death within 2 years from the cardiovascular event both in training and validation sets (AUC 0.837 and 0.797, respectively); age showed comparable results (AUC 0.828 and 0.747) and the GRACE score presented slightly worse results (AUC 0.809 and 0.75).

The performance of our approach was also tested by dividing patients in two distinct subgroups of patients according to AMI severity (ST-segment elevation myocardial infarction (STEMI) and non-STEMI). The results demonstrated that our approach is marginally affected by these two subcategories (Additional file 1: Supplementary material and Figure S6).

### Metabolites analysis

An analysis of the NMR spectra was conducted to identify which metabolites were statistically different between patients who died and survived. All patients (training and validation sets) were included in the analysis. The following 23 metabolites were unambiguously assigned and quantified in the spectra: acetate, acetone, alanine, citrate, creatine, creatinine, formate, glucose, glutamate, glutamine, glycine, histidine, isobutyrate, isoleucine, lactate, leucine, mannose, methionine, phenylalanine, proline, tyrosine, valine, and 3-hydroxybutyrate. We observed that patients who died were characterized by significantly (adjusted *p*-value < 0.05) higher levels of 3-hydroxybutyrate, proline, creatinine, acetate, acetone, formate, and mannose, and significantly lower levels of valine and histidine (Additional file 1: Table S2).

Metabolite analyses replicated separately in the training and validation sets are provided in Additional file 1: Figure S7.

### Sex-specific RF models

The presence of sex-specific differences in human metabolism is well known and the NMR metabolomics is sensitive to these differences [41, 42]. Therefore, we decided to build different RF models for women and men to obtain an outcome clusterization and prediction unbiased by sex.

For each sex-specific group using the RF classifier, the metabolomic NOESY one-dimensional profiles of 60 patients who survived and 30 who died were randomly selected for classification. Both sex models showed significant differential clustering, with a good separation of the two outcome groups (Additional file 1: Figure S8a, d).

Using the NOESY NMR spectra of the female and male cohorts separately, the RF classifier discriminated patients who died from those who survived in the training set with an AUC of 0.786 and 0.834, respectively (Additional file 1: Figure S8b, e). Thresholds of ≥ 0.46 for the female cohort and ≥ 0.476 for the male cohort were set in the training set by optimizing accuracy, sensitivity, and specificity. The results for both cohorts are shown in Additional file 1: Table S3.

When applying these models to the validation set of female and male cohorts, we obtained an AUC of 0.782 and 0.821, respectively (Fig. 3c, f). When using the threshold maximized in the training set, we obtained an overall accuracy of 74.7% and 73.4% for predicting the likelihood of outcome in female and male cohorts, respectively (Additional file 1: Table S3).

On analyzing the spectra of the female and male cohorts, we observed that female patients who died were characterized by higher levels of creatinine, whereas male patients who died were characterized by higher levels of 3-hydroxybutyrate, proline, creatinine, and formate, and significantly lower levels of histidine and valine (Additional file 1: Figure S7b, c).

## Discussion

After the acute phase of an AMI, for which management is strictly defined by the European Society of Cardiology Guidelines [43, 44], patients remain at increased risk of secondary atherothrombotic events, including recurrent ACS events and stroke, and continue to face a high risk of premature death not only in the immediate future but also in the following years [45, 46]. For these reasons, the identification of a metabolomic fingerprint able to identify patients who are at increased risk of death might allow clinicians to tailor medical treatments and interventions according to patients’ overall risk: high-risk patients could be targeted with more intensive pharmacological treatments (that is, with the highest tolerated statin dosage or more aggressive antiplatelet treatments), and more intensive follow-up programs could be planned with clinical reevaluation at shorter time intervals (that is, monthly instead of the standard visits at 1, 6, and 12 months). In AMI patients enrolled in the AMI-Florence 2 study, we found a metabolic fingerprint which was able to discriminate patients who died within 2 years from the cardiovascular event from survivors with high accuracy (AUC 0.859), and this result was duplicated in a validation set (AUC 0.801). We also built sex-specific RF models and found that the male model was better able to predict outcomes (male: AUC 0.834; female: AUC 0.786), which was confirmed in the validation set. To the best of our knowledge, this is the first study to assess the capability of a metabolomic assay to predict mortality in the setting of AMI.

A metabolic fingerprint can be deemed as a holistic super-biomarker with a discriminative and predictive power undoubtedly higher than that of the sum of the few quantified metabolites [47]. AMI, as with the majority of human diseases, has a multifactorial etiology and a complex physiopathology that concurrently alters several metabolic pathways [48]. Therefore, the metabolic fingerprint, composed by superimposing all the visible signals of the low and high molecular weight endogenous metabolites, represents an optimal level at which to analyze pathological changes in biological systems [49]; indeed, it takes into account all metabolite variations, even slight ones.

The NOESY RF score, based on the metabolic fingerprint here presented, was independent from the classical clinical parameters and the widely used GRACE score, and achieved better results in predicting all-cause death within 2 years after AMI when considering both Cox models and ROC analyses. It is worth of mentioning that age was a very good predictor of mortality in our dataset; in particular, it proved to be better than even the GRACE score in our training set. Older patients showed the worst clinical conditions, with a higher percentage of heart failure, atrial fibrillation, previous cerebrovascular diseases, and diabetes. These parameters are not included in the GRACE score model, and could explain why age performed very well as a predictor in our cohort.

Although our study has several strengths, including the number of patients studied, the long-term follow-up (2 years), and the analysis replication in a validation set, some limitations should also be mentioned. First, sample collections were done exclusively in the acute phase of the disease, impairing the acquisition of data correlated to the biochemical mechanisms of the transition to the quiescent phase. Owing to the importance of this aspect, further efforts in this direction are required. Second, even though the data were replicated in a validation set, a totally independent cohort for validation is lacking. However, before attempting to replicate these findings in very large multicenter studies, common standard operating procedures are required for sample collection and storage, otherwise samples collected in different centers will not be comparable. Our group is strongly committed to this, and have contributed to the development of the optimal pre-analytical procedures for metabolomics [25]. Finally, NMR is less sensitive than MS (although it is more suitable for metabolic fingerprinting), and thus only a limited number of metabolites have been found to be statistically significant. Specifically, we found that patients who died were characterized by significantly higher levels of 3-hydroxybutyrate, proline, creatinine, acetate, acetone, formate, and mannose, and significantly lower levels of valine and histidine.

Lifestyle and the medication administered to a patient influence the molecular signatures in plasma and serum samples, and the relative metabolite concentrations reflect tissue lesions and organ dysfunctions. In this framework, previous studies have underlined the usefulness of metabolomics of serum and plasma in determining the individual’s disease risk, prognosis, and therapeutic options in different clinical settings [29, 30, 50, 51]. For instance, sera of heart failure patients carries a strong signature of the disease, allowing the estimation of heart failure-related metabolic disturbance and possessing a better prognostic value than conventional biomarkers [13, 51].

In a prospective study of three population-based cohorts from Finland that were free of CVD at baseline [52], a metabolomic analysis evidenced that circulating phenylalanine, monounsaturated fats, and polyunsaturated fatty acids were as strongly predictive of cardiovascular risk as the conventional lipid risk factors, and were markers of CVD onset during a long-term follow-up (more than a decade). In our study, we did not find any role for these metabolites in predicting mortality. However, the different study populations (CVD- free vs. AMI patients) with different lifestyle and dietary habits may explain the different molecular signatures.

The usefulness of the metabolomic approach was also demonstrated in a general cohort of patients at risk for cardiovascular events undergoing cardiac catheterization [53]; at baseline, plasma metabolomic profiles independently predicted cardiovascular death after adjustment for multiple clinical covariates. In this study, a significant predictive role was demonstrated for five metabolite factors (medium-chain acylcarnitines, short-chain dicarboxylacylcarnitines, long-chain dicarboxylacylcarnitines, branched-chain amino acids, and fatty acids). We consistently found in the present study that higher levels of valine were a protective factor in AMI patients. At variance with our study, increased concentrations of branched-chain amino acids levels have been shown in coronary artery patients compared with control subjects [54], and high levels of these essential amino acids significantly correlated with the severity of coronary artery disease (CAD) [55]. However, these studies were case-control studies and did not evaluate CAD patients in the acute phase of the disease. The pathways of the branched amino acids in humans are very complex and it is likely that in ACS patients an altered metabolic pathway for these amino acids represents a prognostic risk factor.

Our study, performed in a large sample population of AMI patients followed for 2 years, provided new data about the role of high 3-hydroxybutyrate circulating levels on post-AMI mortality. A previous study that evaluated the ketone bodies in the urine of five ACS patients demonstrated that ketone bodies, and particularly 3-hydroxybutyrate, were altered during the acute event [56]. Furthermore, high levels of 3-hydroxybutyrate have been associated with high prevalence of heart failure and diabetes [57]. In insulin deficiency, when the release of free fatty acids from adipose tissues exceeds the capacity of the tissues to metabolize them, severe and potentially fatal diabetic ketoacidosis can occur in which levels of 3-hydroxybutyrate in the blood can reach up to 25 mM [58]. A recent study has also demonstrated a significant increase of serum ketone bodies in response to angioplasty-induced ischemia performed in patients with stable angina, and it has been hypothesized that these metabolic changes could be a response to reperfusion oxidative stress and may play a key role in free radical homeostasis during ischemia-reperfusion injury [59]. However, it remains unclear whether the elevation of ketone bodies represents an adaptive mechanism required to maintain cell metabolism or if it actually contributes to disease progression and, thus, the worsening of the prognosis.

Furthermore, it is worth of mentioning that formate has already been proposed as a possible biomarker of ACS [60].

As expected, patients with worst prognosis showed higher level of serum creatinine, a well-known marker of renal insufficiency. It has already been demonstrated that elevated serum levels of creatinine on admission are associated with impaired myocardial flow and poor prognosis for 1-year mortality [61, 62].

Previous studies evidenced an interaction between sex and adverse cardiovascular events in CAD patients [63–65], with myocardial infarction morbidity and mortality higher in women than in men. Consistently, our results demonstrated a higher prevalence of mortality in women than in men (19.4% vs. 12.5%). The excess risk of mortality in women could be ascribed to the differences in cardiovascular risk factor, that is, age, hypertension, diabetes, and co-morbidities prevalence. Accordingly, in our study, women showed a higher median age and higher prevalence of hypertension, atrial fibrillation, and heart failure with respect to men.

Building sex-specific models enabled us to improve the outcome prediction in the male cohort, but not in the female one. Thus, the male metabolic fingerprint seems to show a higher association with cardiovascular mortality than the female one. However, the female cohort was smaller than the male cohort and this may have affected the predictive capability of the model; therefore, larger cohorts of patients are needed to build robust sex-specific models.

## Conclusions

Our data support the usefulness of the metabolomic approach for identifying a more precise risk profile in AMI patients. Metabolomic analysis by NMR enables fast, approachable, and reproducible characterization of the AMI metabolic fingerprint associated with a poor prognosis, improving the cardiovascular risk assessment beyond that achieved by established risk factors, and identifying those patients who need to undergo a very early and aggressive treatment. Thus, metabolomics could represent a valid addition to the already established risk stratification tools. Further, as demonstrated by the combination of the NOESY RF scores with the Grace score, metabolomics, in combination with classical tests, is able to significantly improve risk classification over the two separate scores.

## Additional file

Additional file 1: **Supplementary material.** Expanded methods and results. **Table S1.** Demographic and Clinical Characteristics divided according to gender. **Table S2.** Univariate Metabolites Analyses. **Table S3.** Results for the gender-specific models. **Figure S1.** Flow chart explaining sample exclusion reasons from the NMR metabolomic analysis. **Figure S2.** Metabolite signal deconvolution using our in-house-developed algorithm for quantification. **Figure S3.** Metabolomic profiles for one randomly selected patient from each group of outcomes. **Figure S4.** Discrimination between patients from different centres using PCA. **Figure S5.** Receiver Operating Characteristic Curves and the corresponding Area for the two models. **Figure S6.** Discrimination between NSTEMI and STEMI using RF. **Figure S7.** Metabolite concentrations. **Figure S8.** Gender-specific models and predictions using NOESY1D spectra. (DOCX 3630 kb)

## Abbreviations

ACS: Acute coronary syndrome
AMI: Acute myocardial infarction
AUC: Area under the curve
CAD: Coronary artery disease
CI: Confidence interval
CPMG: Carr-Purcell-Meiboom-Gill
CVDs: Cardiovascular diseases
GRACE: Global Registry of Acute Coronary Events
MS: Mass spectrometry
NMR: Nuclear magnetic resonance
NOESY: Nuclear Overhauser effect spectroscopy
PCI: Percutaneous coronary intervention
RF: Random forest
ROC: Receiver operating characteristics
STEMI: ST-segment elevation myocardial infarction
