# Supplementary material for: NMR-based metabolomics identifies patients at high risk of death within two years after acute myocardial infarction in the AMI-Florence II cohort
Source: BMC Med. 2019 Jan 7;17:3. doi: 10.1186/s12916-018-1240-2 (PMC6323789; doi:10.1186/s12916-018-1240-2)
Supplement: Supplementary file 1 — Supplementary material. Expanded methods and results. Table S1. Demographic and Clinical Characteristics divided according to gender. Table S2. Univariate Metabolites Analyses. Table S3. Results for the gender-specific models. Figure S1. Flow chart explaining sample exclusion reasons from the NMR metabolomic analysis. Figure S2. Metabolite signal deconvolution using our in-house-developed algorithm for quantification. Figure S3. Metabolomic profiles for one randomly selected patient from each group of outcomes. Figure S4. Discrimination between patients from different centres using PCA. Figure S5. Receiver Operating Characteristic Curves and the corresponding Area for the two models. Figure S6. Discrimination between NSTEMI and STEMI using RF. Figure S7. Metabolite concentrations. Figure S8. Gender-specific models and predictions using NOESY1D spectra. (DOCX 3630 kb) [file 12916_2018_1240_MOESM1_ESM.docx]

**ADDITIONAL FILE 1**

**Additional file 1: Supplementary material - Expanded methods and results**

**Study population**

The study population comprised a group of 978 out of 1496 patients admitted to the Coronary Units of the six hospitals (five community hospitals and one University hospital, the Careggi Hospital) of the Florence health district from April 2008 to April 2009, and enrolled in the frame of the Florence Acute Myocardial Infarction-2 (AMI-Florence 2) registry.

The AMI-Florence Registry prospectively included all patients who arrived alive to the emergency departments of one of the six participating hospitals in the Florence health district with a suspected STEMI between March 2000 and February 2001. All patients fulfilling the diagnostic criteria for acute or sub-acute (symptom onset ≤ 24 or > 24 hours, respectively) STEMI were screened for enrolment in the registry, without any exclusion criteria. The AMI-Florence 2 registry is a second-wave survey of the AMI-Florence registry, and was extended to include all cases of suspected AMI arriving alive to any of the six hospitals, with no exclusion criteria (AMI-Florence 2 included both STEMI and NSTEMI cases).

AMI was diagnosed according to criteria established by the European Society of Cardiology [1]. Briefly, AMI was defined as typical rise and gradual fall of troponin, or more rapid rise and fall of CK-MB, defined as > 99% of normal levels (troponin T >0.05 ng/ml; CK-MB >10 ng/ml), with at least one of the following: acute onset of typical ischaemic chest pain; some Q waves in V1-V3 , 30 ms Q waves 1 mm in two contiguous leads; ST- segment elevation or depression in 2 leads ,

0.2 mV in V1-V3, >0.1 mV in other leads. Unstable angina was defined as a history of new-onset, more frequent, more persistent or rest episode of chest pain, without typical changes of myocardial

enzymes and with ECG evidence of myocardial ischaemia (transient ST segment displacement >0.1 mV during chest pain).

All patients underwent coronary angiography performed by the Judkins’ technique and PCI. Before PCI, all patients received a loading dose of 500 mg of acetylsalicylic acid (ASA) and 300 mg of clopidogrel, followed by 100 (15.9%) or 325 mg (84.1%) of ASA daily and 75 mg of clopidogrel daily. The use of glycoprotein (GP) IIb/IIIa inhibitors was at discretion of the operator and was carried out in 48.5% of patients. Unfractioned heparin 70IU/kg was used in all patients during PCI as anticoagulant. The two-year vital status was assessed by consulting the registry office of the city of residence; therefore, mortality analysis was censored at 24 months after AMI or at date of death, whichever occurred earlier. The subjects were classified as having hypertension according to the guidelines of European Society of Hypertension/ European Society of Cardiology [2] or if they reported taking antihypertensive medications, as verified by the physician. Diabetic subjects were defined in agreement with the American Diabetes Association [3] or on the basis of self-report data (if confirmed by medication or chart review). Dyslipidemia was defined according to the Third report of the National Cholesterol Education Program (NCEP-III) [4] or if they reported taking antidyslipidaemic drugs, as verified by the physician. A positive family history was defined as the presence of at least one first-degree relative who had developed CAD before the age of 55 years for men and 65 years for women.

**In-hospital data collection**

A web-based form was used to collect information on demographics, medical history, clinical and ECG characteristics, treatment and outcomes during hospitalization, and prescriptions at discharge. Time intervals from onset of symptoms to hospital admission was recorded. The Coordinating Centre periodically reviewed data for inconsistencies, and, when necessary, queries were sent to local investigators for further checks before data processing. Completeness of enrolment was periodically checked by comparing hospital discharge data for Acute Coronary Syndromes ICD9-CM codes (primary diagnosis: 410.*, 411.* and 413.*, or 427.5 associated with 410.*, 411.*, 413.* as secondary diagnosis; asterisks indicates only three ICD 9 digits, irrespective of the other digits (fourth and fifth)) from hospitals in the area with data of patients actually entered. When a discharge record could not be tracked in the study database, the original clinical record was checked, and, if enrolment criteria were met, the case was eventually included. Therefore, the series enrolled is population-based and fully representative of incident AMI cases in the area over the study period.

**Serum sample collection and storage**

Blood withdrawals were obtained drew by trained medical personnel and, serum samples were obtained by centrifuging blood sample at 2,000 g for 10 min a 4°C and then stored in aliquots at -80°C until analysis.

**NMR sample preparation**

Frozen serum samples were thawed at room temperature and shaken before use. The NMR samples were prepared according to the standard operating procedures[5]. A total of 300 µL of a sodium phosphate buffer (10.05 g Na_2_HPO_4_·7H_2_O; 0.2 g NaN_3_; 0.4 g sodium trimethylsilyl [2,2,3,3-^2^H_4_]propionate (TMSP) in 500 mL of H_2_O with 20% (v/v) ^2^H_2_O; pH 7.4) was added to 300 µL of each serum sample, and the mixture was homogenized by vortexing for 30 s. A total of 450 µL of this mixture was transferred into a 4.25 mm NMR tube (Bruker BioSpin srl) for the analysis.

**NMR spectra acquisition and processing**

One-dimensional 1H NMR spectra for all samples were acquired using a Bruker 600 MHz spectrometer (Bruker BioSpin) operating at 600.13 MHz proton Larmor frequency, and equipped with a 5 mm CPTCI ^1^H-^13^C-^31^P and ^2^H-decoupling cryoprobe including a z axis gradient coil, an automatic tuning-matching (ATM) and an automatic sample changer. A BTO 2000 thermocouple served for temperature stabilization at the level of approximately 0.1 K at the sample. Before measurement, samples were kept for at least 3 minutes inside the NMR probehead, for temperature equilibration at 310 K.

According to standard practice, for each serum samples three one-dimensional 1H NMR spectra with different pulse sequences were acquired:

(i) a standard nuclear Overhauser effect spectroscopy pulse sequence NOESY 1Dpresat (noesygppr1d.comp; Bruker BioSpin) [6], using 64 scans, 98304 data points, a spectral width of 18028 Hz, an acquisition time of 2.7 s, a relaxation delay of 4 s and a mixing time of 0.01 s, was applied to obtain a spectrum in which both signals of low molecular weight metabolites and high molecular weight aggregates (lipids and lipoproteins) are visible.

(ii) a standard spin echo Carr-Purcell-Meiboom-Gill (CPMG) [7] (cpmgpr1d.comp; Bruker BioSpin) pulse sequence, with 64 scans, 73728 data points, a spectral width of 12019 Hz and a relaxation delay of 4 s, was used for the selective observation of low molecular weight metabolites, suppressing signals arising from high molecular weight aggregates.

(iii) a standard diffusion-edited [8] (ledbgppr2s1d.comp; Bruker BioSpin) pulse sequence, using 64 scans, 98304 data points, a spectral width of 18028 Hz and a relaxation delay of 4 s, was applied to suppress the signals of low molecular weight metabolites.

Free induction decays were multiplied by an exponential function equivalent to a 1.0 Hz line-broadening factor before applying Fourier transformation. Transformed spectra were automatically corrected for phase and baseline distortions and calibrated (anomeric glucose doublet at 5.24 ppm) using TopSpin 3.2 (Bruker Biospin srl).

Each 1D spectrum in the range 0.2-10.00 ppm was segmented into 0.02 ppm chemical shift bins and the corresponding spectral areas were integrated using AMIX software (version 3.8.4, Bruker BioSpin). Binning is a mean to reduce the number of total variables, to compensate for subtle signal shifts, and filter noise in the spectra, making the analysis more robust and reproducible [9, 10]. The region between 4.5 and 5.0 ppm containing the residual water signal was removed and the dimension of the system was reduced to 466 bins. The total spectral area was calculated on the remaining bins and total area normalization was carried out on the data prior to pattern recognition.

**Statistical analysis**

Data analyses were performed using the open source software R. For the demographic and baseline characteristics, the t-test was used for comparison between groups and the chi-square test for comparison between categorical variables.

For the multivariate data analyses of the NMR data the group of 978 patients was randomly split into two independent cohorts: a training set constituted by 80 survivor and 40 dead patients [11], and a validation set constituted by all the remaining patients (106 dead patients and 752 survivor patients).

The initial analysis was restricted to the training set and the first step was to establish if serum metabolomic profiles could distinguish between dead and survivor patients within two years from the cardiovascular event, for this purpose, a Random Forest (RF) classifier [12] was built. This algorithm has many strengths: i) it can deal with large numbers of predictor variables simultaneously, ii) it perform well even in presence of complex non-linear interactions [13], iii) it is almost immune from the overfitting due to the total number of variables in the data, iv) it is applicable when there are more variables than samples; v) it is relatively insensitive to noise; vi) it allows visualization of data in a reduced discriminant space using the proximity matrix calculated during the process of forest growing; vii) the percentage of trees in the forest that assign one sample to a specific class can be interpreted as a probability of class belonging; and viii) it gives an unbiased estimate of the classification error using the out-of-bag samples, avoiding the need for time-consuming cross validation.

RF is a classification algorithm that uses an ensemble of unpruned decision trees (forest), each of which is built on a bootstrap sample of the training data using a randomly selected subset of variables [14, 15]. In a typical bootstrap sample, approximately 63% of the original observations occur at least once. Observations in the original dataset that do not happen to occur in a bootstrap sample are defined as out-of-bag observations (OOB). A classification tree is fitted to each bootstrap sample but, at each node, only a small number of randomly selected variables are included in the model. The trees are fully grown, and each is used to predict the OOB observations. The predicted class of an observation is calculated by the majority vote of the OOB predictions for that observation, with ties split randomly. Accuracies and error rates are computed for each observation using OOB predictions, and then averaged over all observations. Because the OOB observations were not used in the fitting of the trees, the OOB estimates are cross-validated accuracy estimates, and represent an unbiased estimation of the generalization error [16]. The percentage of trees in the forest that assign one sample to a specific class can be inferred as a probability of class belonging [17].

In our case, each tree is used to predict whether a sample is associated with positive or negative outcome (alive or dead patients). For each patient, a score was created that expresses the extent to which the serum metabolomic profile appears to be similar to the one of dead patients, designated as the ‘RF risk score’. This score is based on the percentage of trees in the ensemble that misclassify the sample as belonging to the cohort of dead patients. For each patient, three RF scores were derived using the three types of spectra acquired. For all calculations, the R package ‘Random Forest’ [12] was used to grow a forest of 2000 trees, using the default settings.

The next step was to test the hypothesis that a metabolomic signature similar to the one of patients with a negative outcome would be really predictive of death within two years from the CV event. Using receiver operating characteristics (ROC) analysis (“colAUC” function of the R package “caTools”) and Harrell's c index (“cindex” function on the R package “dynpred”), the performance of the RF risk scores were compared with the actual outcome. To delineate high risk of death, a cut-off for the RF risk score was calculated in the training set that optimized accuracy, sensitivity and specificity, and the performance of the model was subsequently tested in the validation set. Sensitivity, specificity, and accuracy were calculated according to the standard definition of, respectively: proportion of dead patients that are correctly identified as such with respect to the total death patients; proportion of alive patients that are correctly identified as such with respect to the total alive patients; proportion of correctly classified individuals (both dead and alive) that are correctly identified with respect to the total cohort.

**Exploratory unsupervised analysis of the dataset**

Principal Component Analysis (PCA) was performed as first exploratory analysis. It is apparent that no unsupervised discrimination between the main hospital (Careggi University Hospital) and the other centers is present. The score plot of the first two principal components is provided (Additional file 1: Figure S4).

**Distinct metabolomic models calculated according to AMI severity**

Patients enrolled in this study were 378 STEMI and 600 NSTEMI. Using a Random Forest classifier it is possible to discriminate between these two subgroups with 66.5% accuracy (Additional file 1: Figure S6), indicating that the serum metabolic fingerprint is weakly affected by the AMI severity. The possible implications of these differences due to sub-phenotypes of STEMI and NSTEMI has been further investigated by building two distinct models for the outcome prediction: 100 cycles of classical Monte Carlo cross-validation scheme with 80-20 % splitting of the data (training set, validation set) have been performed for each sub-group, and a mean AUC of 0.829 (95% CI, 0.812-0.846) and of 0.784 (95% CI, 0.774-0.794) were obtained for STEMI and NSTEMI, respectively. Comparing these results with the mean cross-validated AUC obtained using all the dataset together (AUC 0.805), we can conclude that the division of patients according to severity contributes only marginally to the metabolomic fingerprint associated with the outcome, and, as a consequence, that the prognostic value of the metabolic fingerprint reported here is not just a proxy of the clinical status, but a real instrument able to improve the risk stratification in the framework of post ACS patients.

**Additional Tables**

|  | **Male** | | **Female** | | | **P-value**  **M *vs* F** |
| --- | --- | --- | --- | --- | --- | --- |
|  | **Survivor patients (554)** | **Dead patients (79)** | | **Survivor patients (278)** | **Dead patients (67)** |  |
| **Age (years), median (IQR)** | 70 (59-77) | 81 (76-87) | | 77 (70-84) | 83 (80-88) | 2.2·10^-16^ |
| **Cardiovascular risk factors, n (%)** |  |  | |  |  |  |
| Hypertension | 340 (61.4) | 52 (65.8) | | 197 (70.9) | 52 (77.6) | 1.15·10^-03^ |
| Dyslipidemia | 191 (29.0) | 18 (22.8) | | 103 (37.1) | 14 (20.9) | 7.78·10^-01^ |
| Current smokers | 186 (33.6) | 15 (19.0) | | 40 (14.4) | 5 (7.5) | 1.46·10^-20^ |
| Ex-smokers | 13 (2.9) | 5 (6.3) | | 8 (2.9) | 3 (4.5) | 1.09·10^-01^ |
| CAD | 160 (28.9) | 9 (11.4) | | 60 (21.6) | 8 (2.9) | 3.14·10^-02^ |
| Diabetes | 121 (21.8) | 35 (44.3) | | 76 (27.3) | 26 (38.8) | 1.21·10^-01^ |
| **Medical history, n (%)** |  |  | |  |  |  |
| Myocardial infarction | 109 (19.7) | 32 (40.5) | | 55 (19.8) | 16 (23.9) | 5.51·10^-01^ |
| Angina, onset > 1 month | 76 (13.7) | 14 (17.7) | | 43 (15.5) | 10 (14.9) | 1.00·10^+00^ |
| Angina, onset ≤ 1 month | 108 (19.5) | 9 (11.4) | | 41 (14.7) | 6 (9.0) | 6.27·10^-02^ |
| CABG | 31 (5.6) | 7 (8.9) | | 10 (3.6) | 3 (4.5) | 1.71·10^-01^ |
| PCI | 96 (17.3) | 23 (29.1) | | 40 (14.4) | 9 (13.4) | 7.77·10^-02^ |
| Chronic heart failure | 17 (3.1) | 11 (13.9) | | 16 (5.8) | 15 (22.4) | 6.73·10^-03^ |
| Atrial fibrillation | 19 (3.4) | 8 (10.1) | | 23 (8.3) | 13 (19.4) | 3.20·10^-04^ |
| Cerebrovascular disease | 31 (5.6) | 14 (17.7) | | 19 (6.8) | 14 (20.9) | 2.24·10^-01^ |
| **Presentation features** |  |  | |  |  |  |
| ACS classification, STEMI, n (%) | 250 (45.1) | 17 (21.5) | | 93 (33.4) | 18 (26.9) | 2.68·10^-03^ |
| Killip II-IV, n (%) | 59 (10.7) | 29 (36.7) | | 53 (19.1) | 32 (47.8) | 2.16·10^-05^ |
| Creatinine > 1.2 mg/dL, n (%) | 85 (15.3) | 32 (40.5) | | 44 (15.8) | 22 (32.8) | 1.00·10^+00^ |
| Heart rate (bpm), median (IQR) | 77.5 (65-89) | 89 (80-100) | | 83 (70-98) | 94 (80-106) | 3.36·10^-05^ |
| Peak troponine max, n (%) | 532 (96.0) | 78 (98.7) | | 272 (97.8) | 65 (97.0) | 1.00·10^+00^ |
| Peak CK-MB max, n (%) | 297 (53.6) | 31 (39.2) | | 125 (45.0) | 19 (28.4) | 2.89·10^-03^ |

**Additional file 1: Table S1.** Demographic and Clinical Characteristics divided according to gender

CAD indicates coronary artery diseases; CABG, coronary artery bypass grafting; PCI, percutaneous coronary intervention; ACS, acute coronary syndrome; STEMI, ST-segment elevation myocardial infarction; CK-MB, creatine kinase-MB; GRACE, Global Registry of Acute Coronary Events

**Additional file 1: Table S2.** Univariate Metabolites Analyses.

| **Assigned metabolites** | **Dead cohort** | **Survivor cohort** | **Adjusted *P*-value** | **Effect size (Cliff's delta)** | **Fold change** |
| --- | --- | --- | --- | --- | --- |
| Acetate | 97.14 ± 66.16 | 78.27 ± 47.94 | 3.10·10^-02^ | 0.167 | 0.312 |
| Acetone | 913.9 ± 819.93 | 747.66 ± 564.49 | 4.10·10^-02^ | 0.163 | 0.290 |
| Alanine | 1384.39 ± 319.37 | 1499.65 ± 368.9 | 1.32·10^-01^ | -0.144 | -0.115 |
| Citrate | 100.18 ± 39.36 | 89.58 ± 30.12 | 1.82·10^-01^ | 0.138 | 0.161 |
| Creatine | 101.15 ± 78.45 | 103.11 ± 60.2 | 1.00·10^+00^ | 0.016 | -0.028 |
| Creatinine | 265.11 ± 107.24 | 198.4 ± 56.18 | 1.48·10^-11^ | 0.373 | 0.418 |
| Formate | 11.04 ± 3.5 | 9.48 ± 3.26 | 1.46·10^-04^ | 0.234 | 0.221 |
| Glucose | 3034.26 ± 1143.89 | 2758.32 ± 755.37 | 1.79·10^-01^ | 0.139 | 0.138 |
| Glutamate | 175.21 ± 108.52 | 205.48 ± 106.61 | 1.00·10^+00^ | -0.099 | -0.230 |
| Glutamine | 201.64 ± 66.5 | 189.8 ± 45.97 | 1.00·10^+00^ | 0.103 | 0.087 |
| Glycine | 494.25 ± 170.9 | 528.65 ± 159.84 | 1.00·10^+00^ | -0.079 | -0.097 |
| Histidine | 106.22 ± 21.98 | 114.64 ± 22.04 | 4.92·10^-04^ | -0.221 | -0.110 |
| Isobutyrate | 35.54 ± 14.59 | 30.36 ± 12.47 | 9.05·10^-02^ | 0.150 | 0.227 |
| Isoleucine | 150.73 ± 41.74 | 163.78 ± 42.15 | 1.11·10^-01^ | -0.147 | -0.120 |
| Lactate | 1594.03 ± 575.05 | 1536.27 ± 419.58 | 1.00·10^+00^ | 0.007 | 0.053 |
| Leucine | 482.98 ± 151.7 | 523.63 ± 120 | 9.86·10^-01^ | -0.106 | -0.117 |
| Mannose | 122.14 ± 54.41 | 105.44 ± 34.71 | 8.33·10^-03^ | 0.185 | 0.212 |
| Methionine | 115.76 ± 42.64 | 110.98 ± 35.8 | 1.00·10^+00^ | 0.058 | 0.061 |
| Phenylalanine | 234.35 ± 65.27 | 228.57 ± 59.25 | 8.20·10^-01^ | 0.110 | 0.036 |
| Proline | 158.42 ± 94.5 | 114.52 ± 59.5 | 2.97·10^-07^ | 0.295 | 0.468 |
| Tyrosine | 167.18 ± 44.94 | 170.61 ± 37.32 | 1.00·10^+00^ | -0.043 | -0.029 |
| Valine | 1016.1 ± 250.83 | 1127.15 ± 237.85 | 4.69·10^-06^ | -0.270 | -0.150 |
| 3-hydroxybutyrate | 488.44 ± 526.3 | 319.89 ± 310.27 | 1.17·10^-03^ | 0.211 | 0.611 |

List of metabolites assigned in the serum NMR spectra, reported as median with median absolute deviation (arbitrary units). A *P*-value adjusted with Bonferroni < 0.05 deemed significant. The Cliff’s delta and the fold change for each metabolite are also reported.

**Additional file 1: Table S3.** Results for the gender-specific models

|  | **Male cohort** | **Female cohort** |
| --- | --- | --- |
| **Training set, % (95% CI)** |  |  |
| Sensitivity | 83.3% (95% CI 82.6-83.6%) | 84.6% (95% CI 84.2-84.9%) |
| Specificity | 74.2% (95% CI 73.2-75.2%) | 70.0% (95% CI 68.7-71.3%) |
| Accuracy | 77.2% (95% CI 76.5-77.9%) | 74.9% (95% CI 74.0-75.7%) |
| **Validation set, %** |  |  |
| Sensitivity | 73.1% | 68.8% |
| Specificity | 73.5% | 75.7% |
| Accuracy | 73.4% | 74.7% |

CI = confidence interval

**Additional Figures**

**

Additional file 1: Figure S1.** Flow chart explaining sample exclusion reasons from the NMR metabolomic analysis.

**Additional file 1: Figure S2.** Upper: The NMR spectrum region with the above study metabolite annotated. Below: Our algorithm metabolites NMR signals deconvolution: all NMR peaks in the above study NMR spectral region are fitted and the fitting procedure residuals are identified. Finally, metabolite NMR peaks of our interest are extracted and integrated, resulting to the metabolite quantification with high level of accuracy. As examples A) acetone; B) phenylalanine.

A)


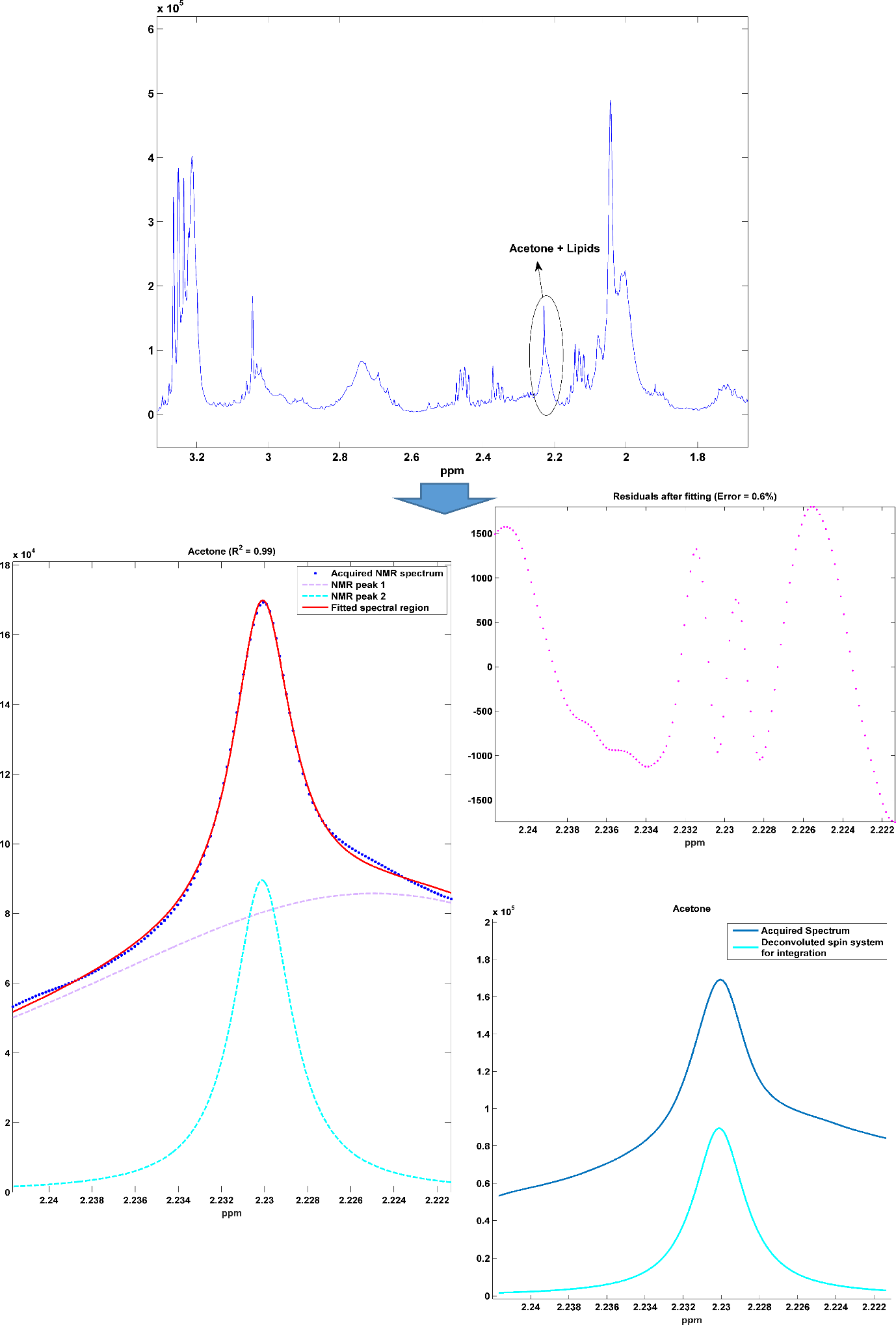


B)


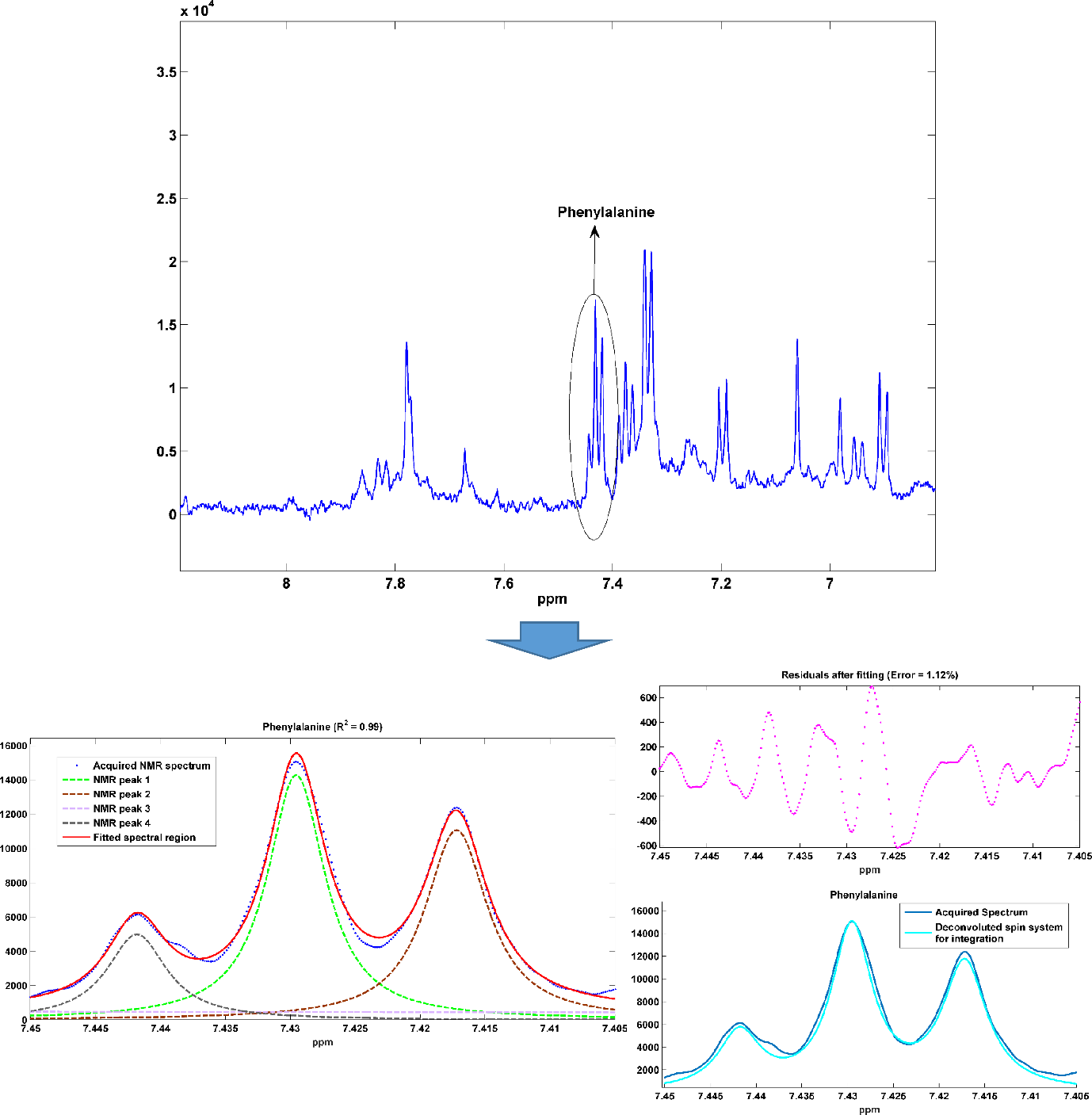


**Additional file 1: Figure S3.**  Metabolomic profiles for one randomly selected patient from each group of outcomes. For each patients NOESY, CPMG, and Diffusion spectra are displayed.

**
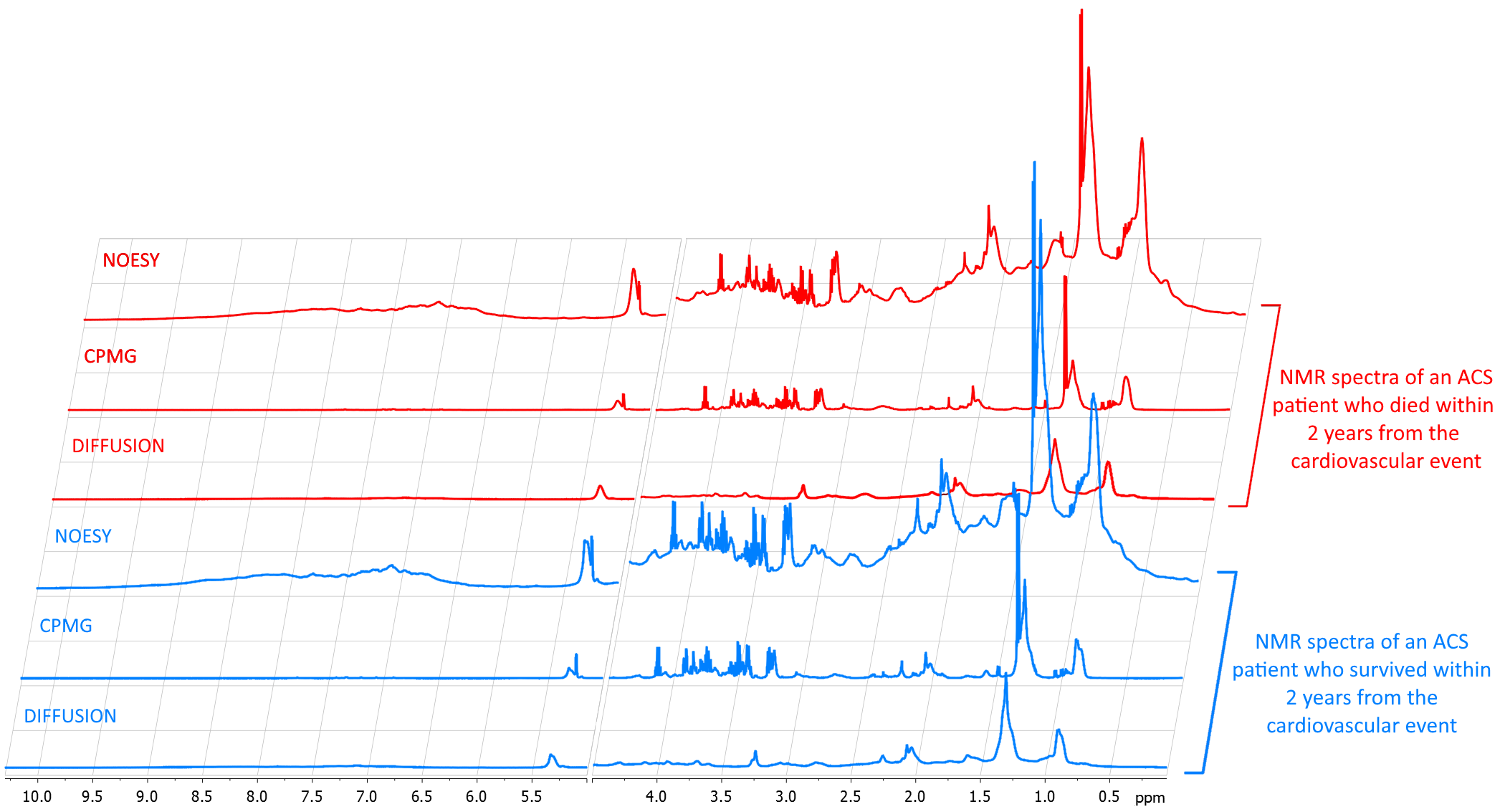
**

**Additional file 1: Figure S4.** Discrimination between Careggi University Hospital (blue dots, n = 579) and other centers (orange dots, n = 399) using Principal Component Analysis.


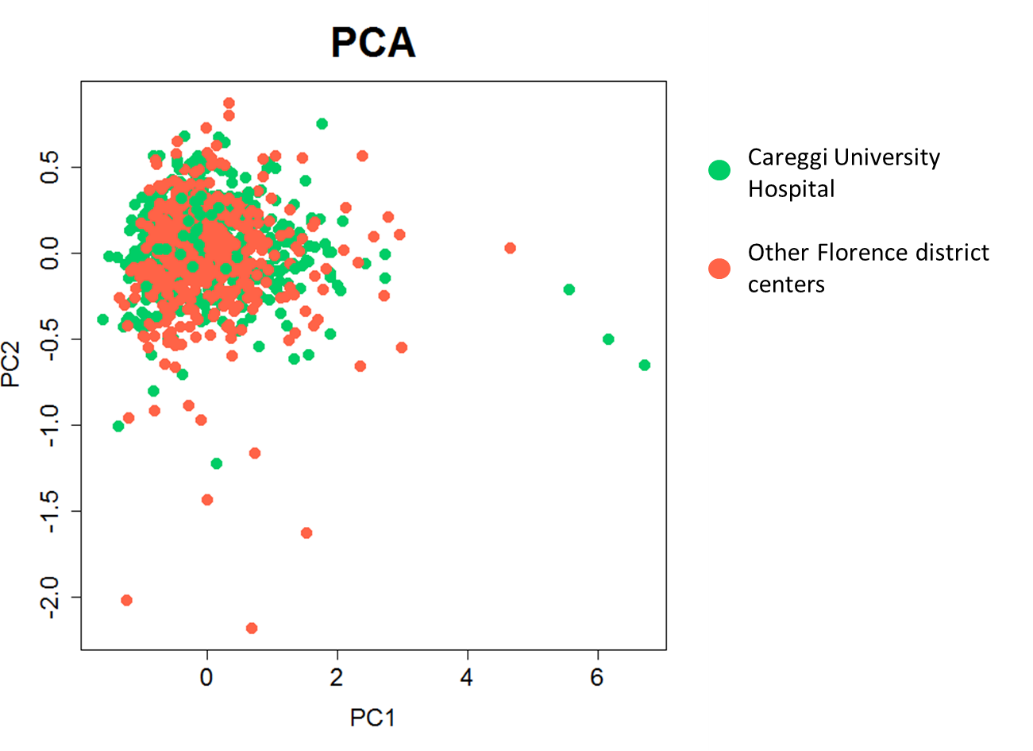


**Additional file 1: Figure S5.** Receiver Operating Characteristic Curves and the corresponding Area are reported for two models: Model 1, A) Training set: Careggi University Hospital; B) Validation set: Other Hospitals. Model 2, C) Training set: Other Hospitals; B) Validation set: Careggi University Hospital.


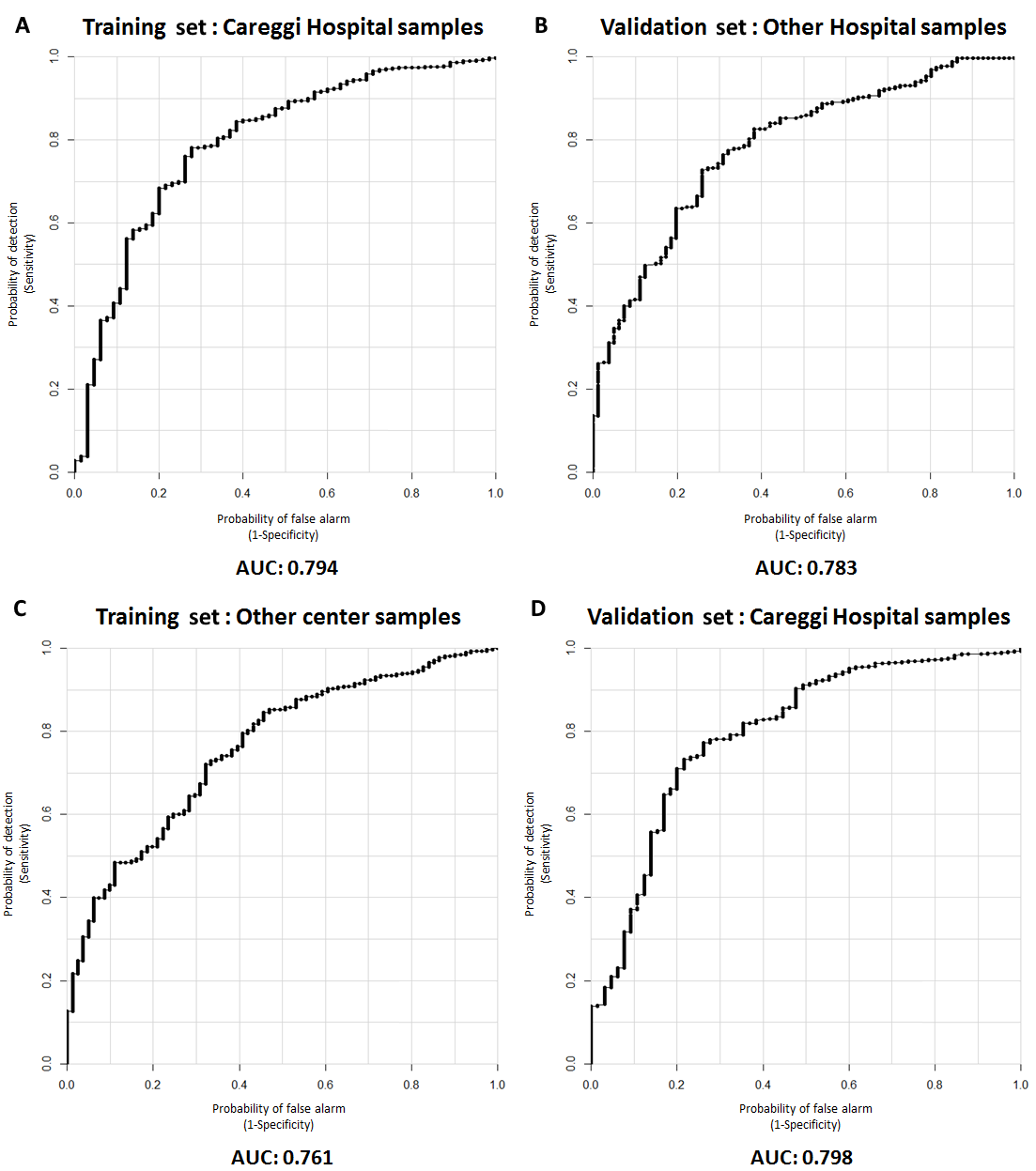


**Additional file 1: Figure S6.** Discrimination between NSTEMI (blue dots, n = 600) and STEMI (red dots, n = 378) patients using the Random Forest. Discrimination accuracy is 66.5%.


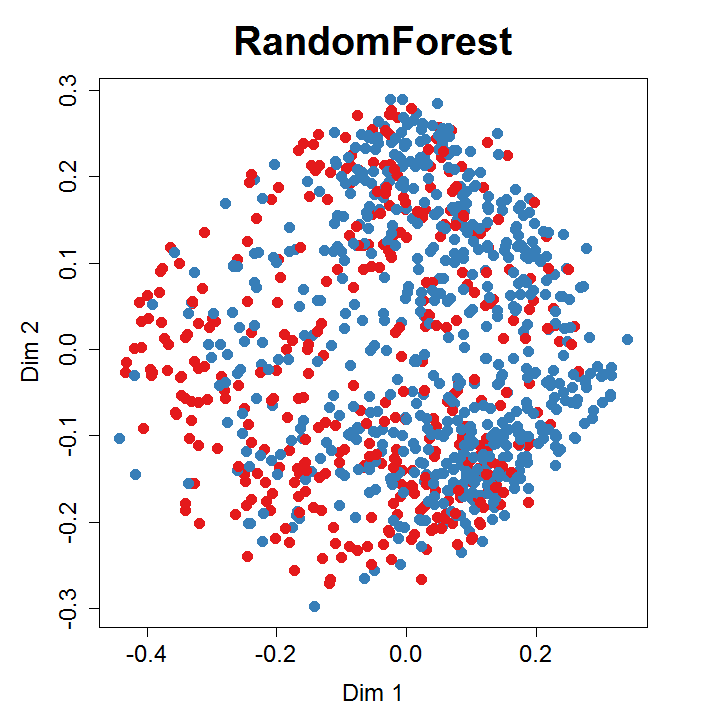


**Additional file 1: Figure S7.** Metabolite concentrations in: A) All dataset; B) Training set; C) Validation set. Metabolites are reported as log2 of the Fold Change, and positive values mean higher concentrations in dead patients. Green bars represent statistically significant *P*-values (P<0.05 after correction), conversely red bars mean not significant *P*-values.


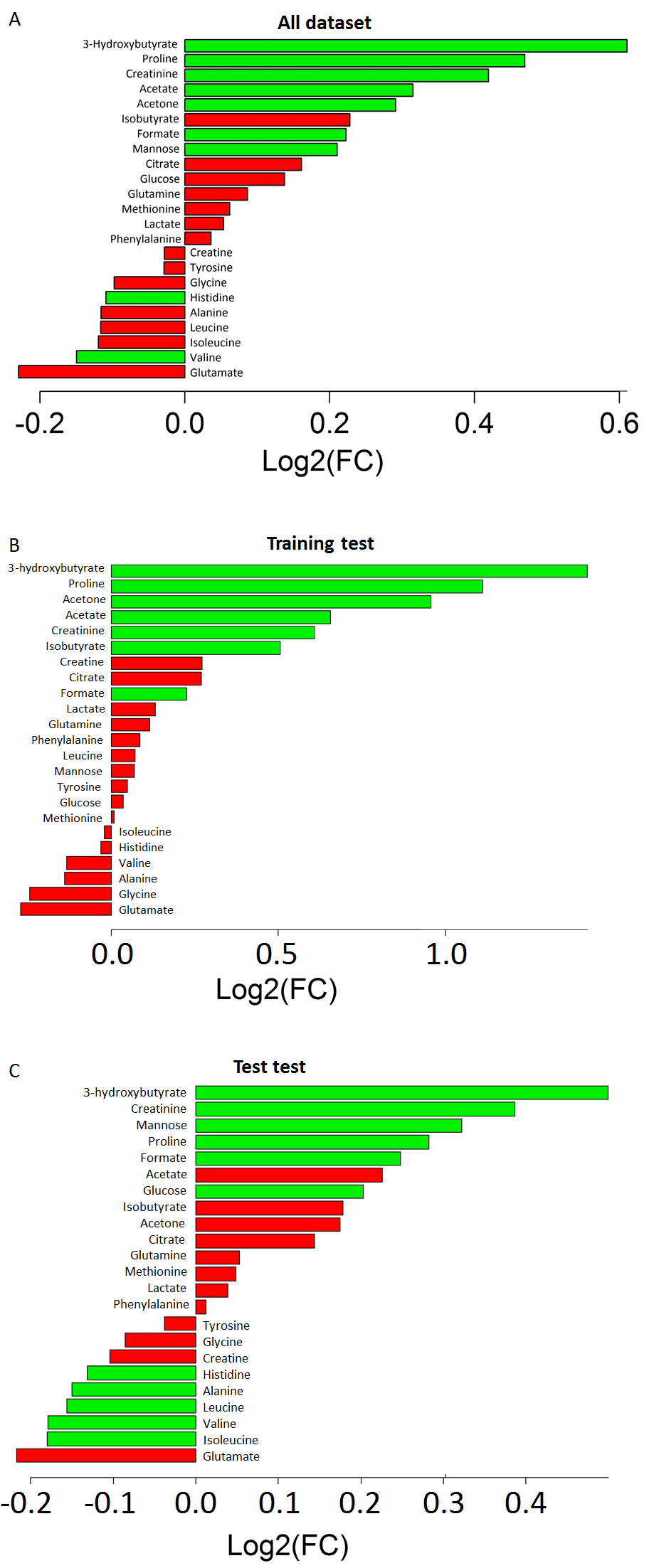


**Training set**

**Validation set**


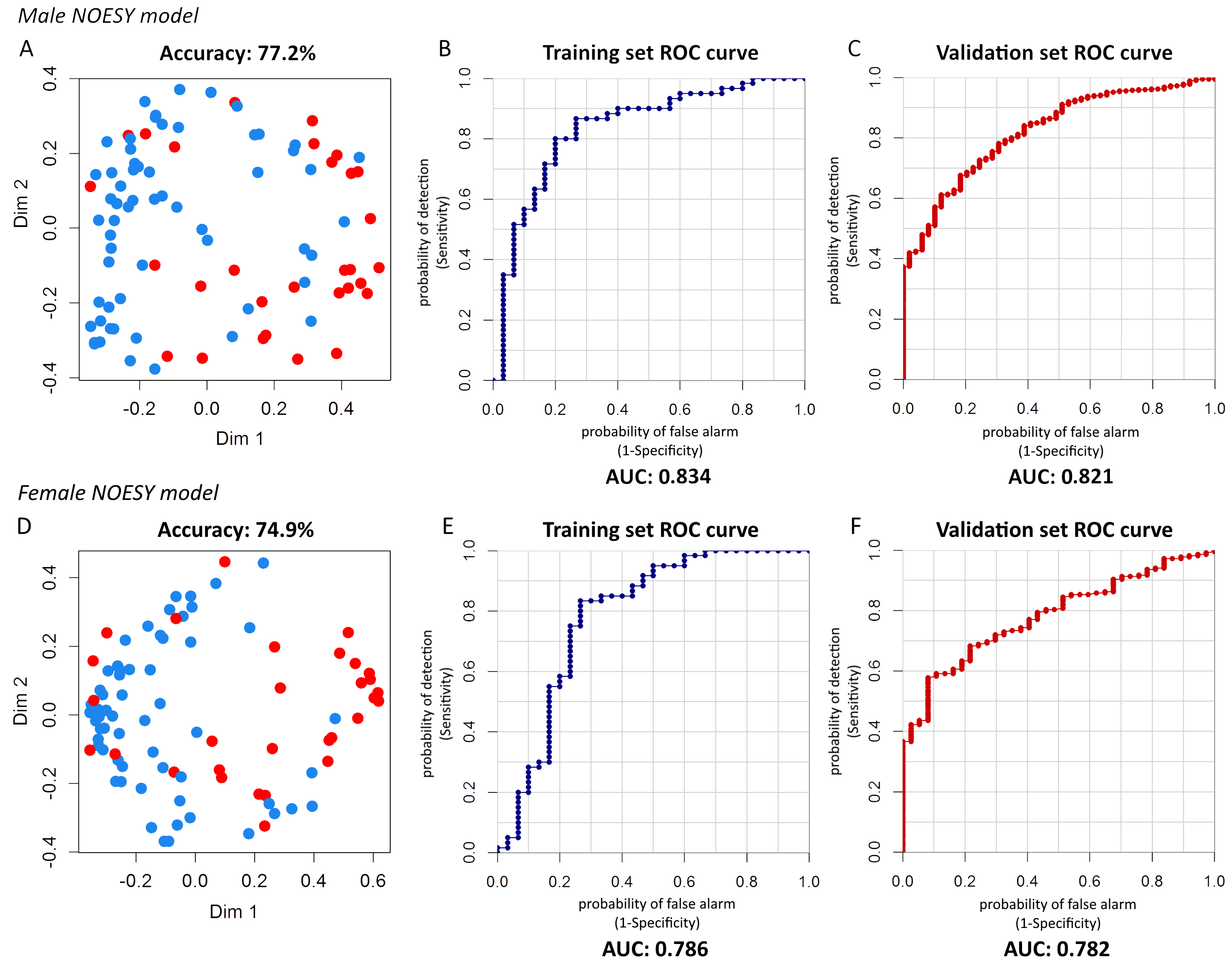
 **Additional file 1: Figure S8.** Gender-specific models and predictions using NOESY1D spectra. Clusterization of serum metabolomic profiles discriminating between survivor (blue dots) and dead (red dots) patients using the Random Forest classifier for A) male; D) female. Comparison between metabolomic classification and outcomes in the training set: the ROC curves and the AUC scores are presented for B) male; E) female. Prediction of outcomes in the validation set: the ROC and the AUC scores are presented for C) male; F) female.

**References**

1. Hamm CW, Bassand J-P, Agewall S, Bax J, Boersma E, Bueno H, et al. ESC Guidelines for the management of acute coronary syndromes in patients presenting without persistent ST-segment elevation: The Task Force for the management of acute coronary syndromes (ACS) in patients presenting without persistent ST-segment elevation of the European Society of Cardiology (ESC). Eur Heart J. 2011;32:2999–3054.

2. Cifkova R, Erdine S, Fagard R, Farsang C, Heagerty AM, Kiowski W, et al. Practice guidelines for primary care physicians: 2003 ESH/ESC hypertension guidelines. J Hypertens. 2003;21:1779–86.

3. Expert Committee on the Diagnosis and Classification of Diabetes Mellitus. Report of the expert committee on the diagnosis and classification of diabetes mellitus. Diabetes Care. 2003;26 Suppl 1:S5-20.

4. Wilkins LW&. Third Report of the National Cholesterol Education Program (NCEP) Expert Panel on Detection, Evaluation, and Treatment of High Blood Cholesterol in Adults (Adult Treatment Panel III) Final Report. Circulation. 2002;106:3143–3143.

5. Bernini P, Bertini I, Luchinat C, Nincheri P, Staderini S, Turano P. Standard operating procedures for pre-analytical handling of blood and urine for metabolomic studies and biobanks. J Biomol NMR. 2011;49:231–43.

6. Mckay RT. How the 1D-NOESY suppresses solvent signal in metabonomics NMR spectroscopy: An examination of the pulse sequence components and evolution. Concepts Magn Reson Part A. 2011;38A:197–220.

7. Meiboom S, Gill D. Modified Spin‐Echo Method for Measuring Nuclear Relaxation Times. Rev Sci Instrum. 1958;29:688–91.

8. Wu DH, Chen A. Three-dimensional diffusion-ordered NMR spectroscopy: The homonuclear COSY-DOSY experiment. J Magnen Reson A. 1996;123:215–8.

9. Spraul M, Neidig P, Klauck U, Kessler P, Holmes E, Nicholson JK, et al. Automatic reduction of NMR spectroscopic data for statistical and pattern recognition classification of samples. J Pharm Biomed Anal. 1994;12:1215–25.

10. Holmes E, Foxall PJ, Nicholson JK, Neild GH, Brown SM, Beddell CR, et al. Automatic data reduction and pattern recognition methods for analysis of 1H nuclear magnetic resonance spectra of human urine from normal and pathological states. Anal Biochem. 1994;220:284–96.

11. Kotsiantis S, Kanellopoulos D, Pintelas P. Handling imbalanced datasets: A review. GESTS Int Transter Sci Eng CompugResearchGate. 2005;30:25–36.

12. Breiman L. Random Forests. Mach Learn. 2001;45:5–32.

13. Strobl C, Malley J, Tutz G. An introduction to recursive partitioning: rationale, application, and characteristics of classification and regression trees, bagging, and random forests. Psychol Methods. 2009;14:323–48.

14. Touw WG, Bayjanov JR, Overmars L, Backus L, Boekhorst J, Wels M, et al. Data mining in the Life Sciences with Random Forest: a walk in the park or lost in the jungle? Brief Bioinform. 2013;14:315–26.

15. Verikas A, Gelzinis A, Bacauskiene M. Mining data with random forests: A survey and results of new tests. Pattern Recognit. 2011;44:330–49.

16. Bertini I, Luchinat C, Miniati M, Monti S, Tenori L. Phenotyping COPD by 1H NMR metabolomics of exhaled breath condensate. Metabolomics. 2013;10:302–11.

17. Tenori L, Oakman C, Morris PG, Gralka E, Turner N, Cappadona S, et al. Serum metabolomic profiles evaluated after surgery may identify patients with oestrogen receptor negative early breast cancer at increased risk of disease recurrence. Results from a retrospective study. Mol Oncol. 2015;9:128–39.
